# Supplementary material for: Determining propensity for sub-optimal low-density lipoprotein cholesterol response to statins and future risk of cardiovascular disease
Source: PLoS One. 2021 Dec 2;16(12):e0260839. doi: 10.1371/journal.pone.0260839 (PMC8638964; doi:10.1371/journal.pone.0260839)
Supplement: S1 Fig — Validation plots for logistic model applied in (a) UK CPRD internal validation cohort, n = 54,985 and (b) HK CDARS external validation cohort, n = 170,904. E:O–log of the expected/observed number of events; CITL–calibration-in-the-large; AUC–area under the curve; slope–calibration slope. The circles represent deciles of patients grouped by similar predicted risk. The distribution of patients (stratified by outcome) is indicated with spikes at the bottom of the graph. Patients with sub-optimal response are represented by spikes above the x-axis (red line), and patients with optimal response, below the x-axis). (DOCX) [file pone.0260839.s001.docx]

**S1 Figure. Validation plots for logistic model applied in (a) UK CPRD internal validation cohort, n=54,965 and (b) HK CDARS external validation cohort, n=170,904.**


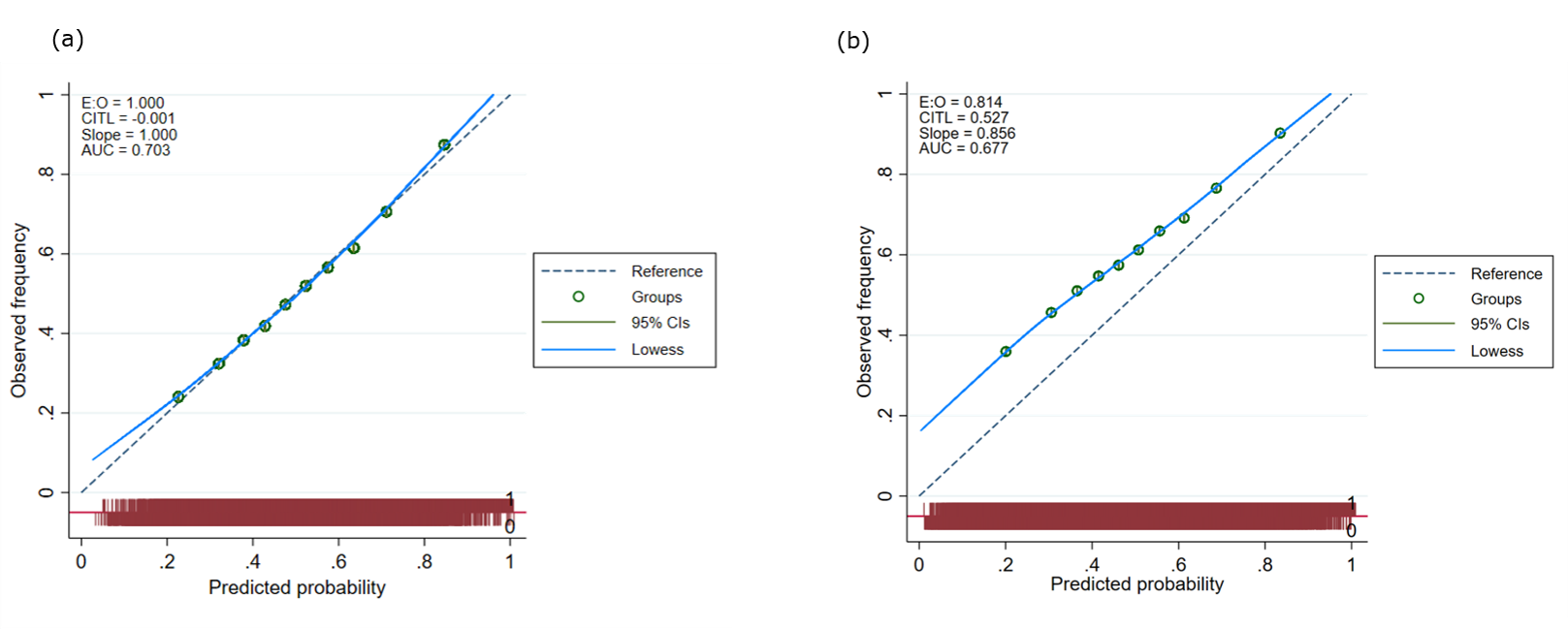


Hosmer-Lemeshow $\chi^{2}$ = 0.93, p = 0.630 Hosmer-Lemeshow $\chi^{2}$ = 10142, p = 0.000

E:O – log of the expected/observed number of events; CITL – calibration-in-the-large; AUC – area under the curve; slope – calibration slope.

The circles represent deciles of patients grouped by similar predicted risk. The distribution of patients (stratified by outcome) is indicated with spikes at the bottom of the graph. Patients with sub-optimal response are represented by spikes above the x-axis (red line), and patients with optimal response, below the x-axis).
