# Supplementary material for: Determining propensity for sub-optimal low-density lipoprotein cholesterol response to statins and future risk of cardiovascular disease
Source: PLoS One. 2021 Dec 2;16(12):e0260839. doi: 10.1371/journal.pone.0260839 (PMC8638964; doi:10.1371/journal.pone.0260839)

**S2 Figure. Plot of sensitivity and specificity to determine optimum cut-off for the classification of sub-optimal LDL response risk to statins using the standard approach model in the UK CPRD validation cohort (n=54,965)**


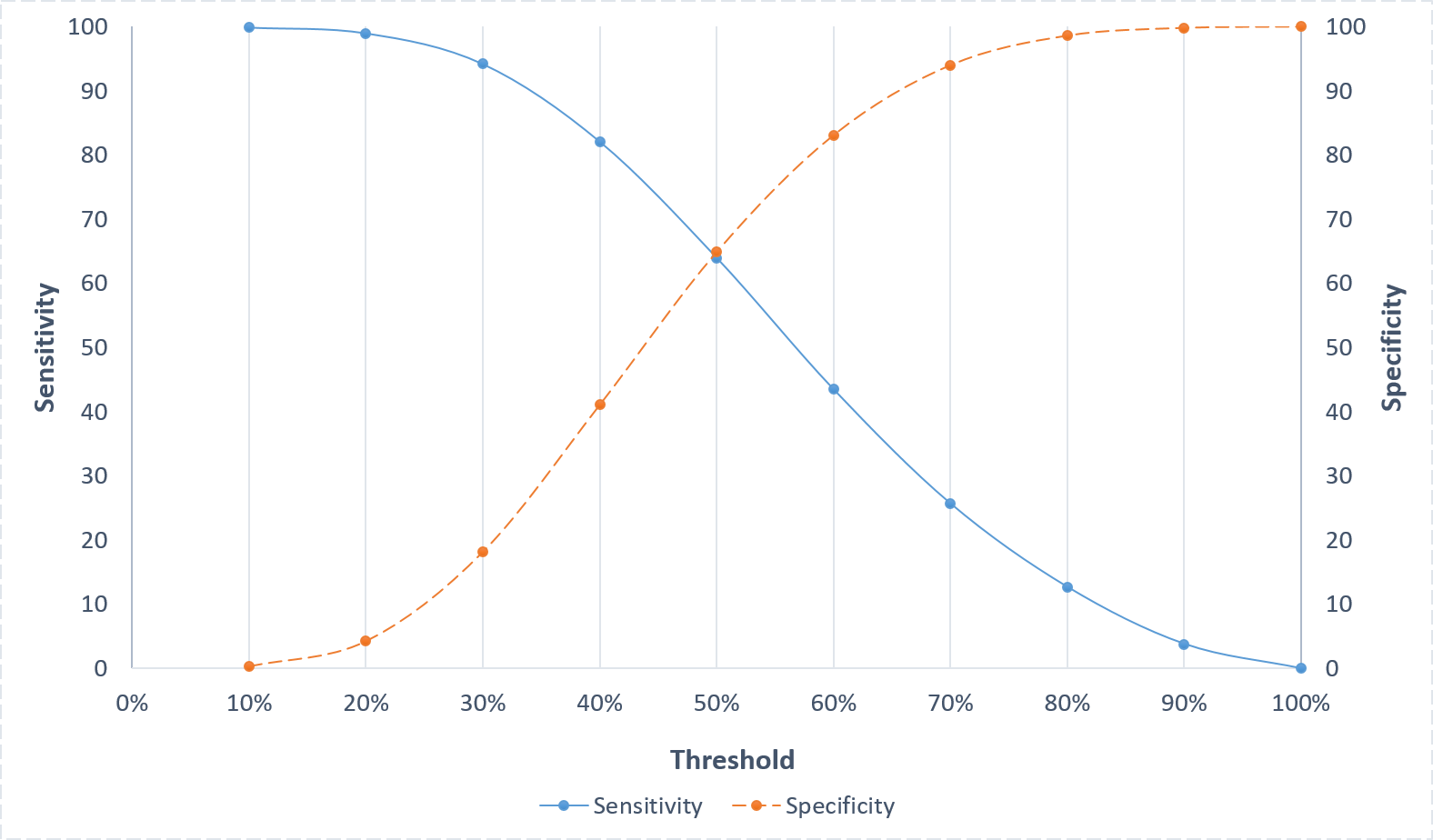

Supplement: S2 Fig — (DOCX) [file pone.0260839.s002.docx]
