# Supplementary material for: Determining propensity for sub-optimal low-density lipoprotein cholesterol response to statins and future risk of cardiovascular disease
Source: PLoS One. 2021 Dec 2;16(12):e0260839. doi: 10.1371/journal.pone.0260839 (PMC8638964; doi:10.1371/journal.pone.0260839)

**S3 Figure. Histograms of predicted 10-year CVD risk score by ESC guidelines and 2-year propensity of sub-optimal LDL-C reduction for UK and HK cohorts**


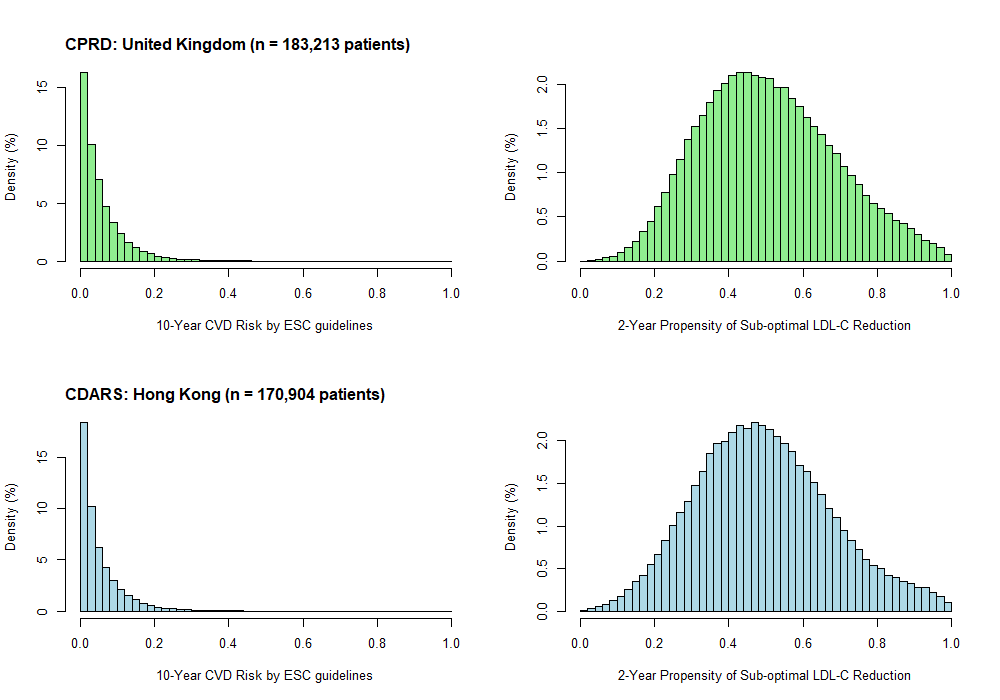

Supplement: S3 Fig — (DOCX) [file pone.0260839.s003.docx]
