# Supplementary material for: Determining propensity for sub-optimal low-density lipoprotein cholesterol response to statins and future risk of cardiovascular disease
Source: PLoS One. 2021 Dec 2;16(12):e0260839. doi: 10.1371/journal.pone.0260839 (PMC8638964; doi:10.1371/journal.pone.0260839)
Supplement: S4 Fig — ESC: European Society of Cardiology CVD SCORE >5% is defined as high CVD risk; Low-density lipoprotein cholesterol statin response propensity of > 50% is defined as sub-optimal response. (DOCX) [file pone.0260839.s004.docx]

**S4 Figure. Joint stratification of 10-year CVD risk by ESC guidelines and 2-year propensity of sub-optimal LDL-C reduction in both UK and HK patient cohorts.**


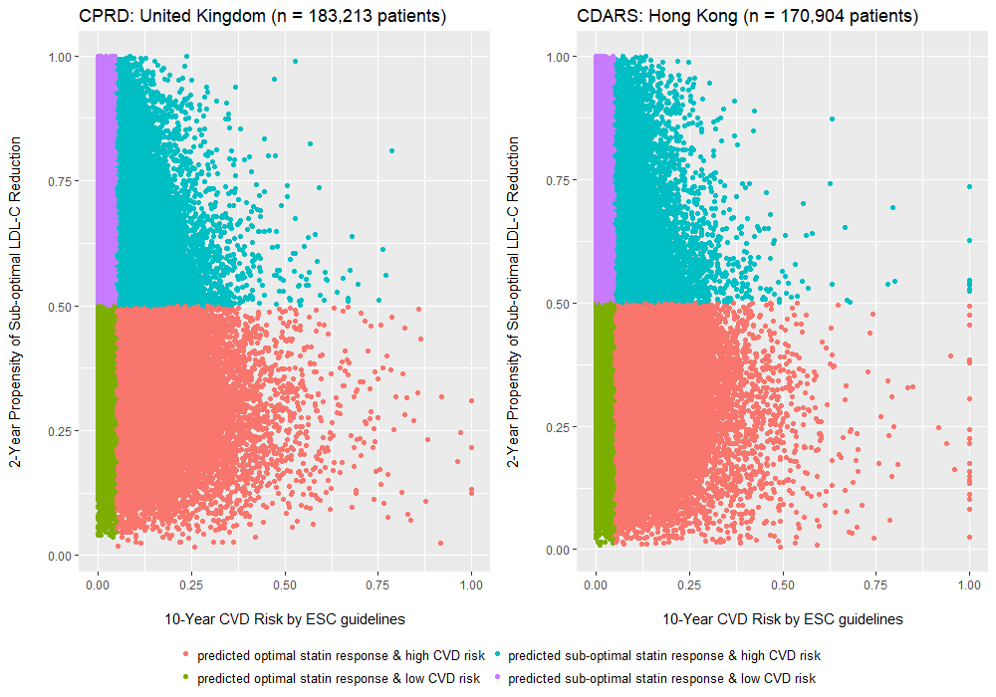


ESC: European Society of Cardiology CVD SCORE >5% is defined as high CVD risk; Low-density lipoprotein cholesterol statin response propensity of > 50% is defined as sub-optimal response.
