# Supplementary material for: Determining propensity for sub-optimal low-density lipoprotein cholesterol response to statins and future risk of cardiovascular disease
Source: PLoS One. 2021 Dec 2;16(12):e0260839. doi: 10.1371/journal.pone.0260839 (PMC8638964; doi:10.1371/journal.pone.0260839)
Supplement: S5 Fig — Potency classification by expected LDL-C reduction (see S2 Table for specific statin types); 20%–30%: low potency; 31%–40%: medium potency; Above 40%: high potency. (DOCX) [file pone.0260839.s005.docx]

**S5 Figure.** **Waterfall plots of 2-year percentage LDL-C reduction from baseline based on initiated statin potency**

**
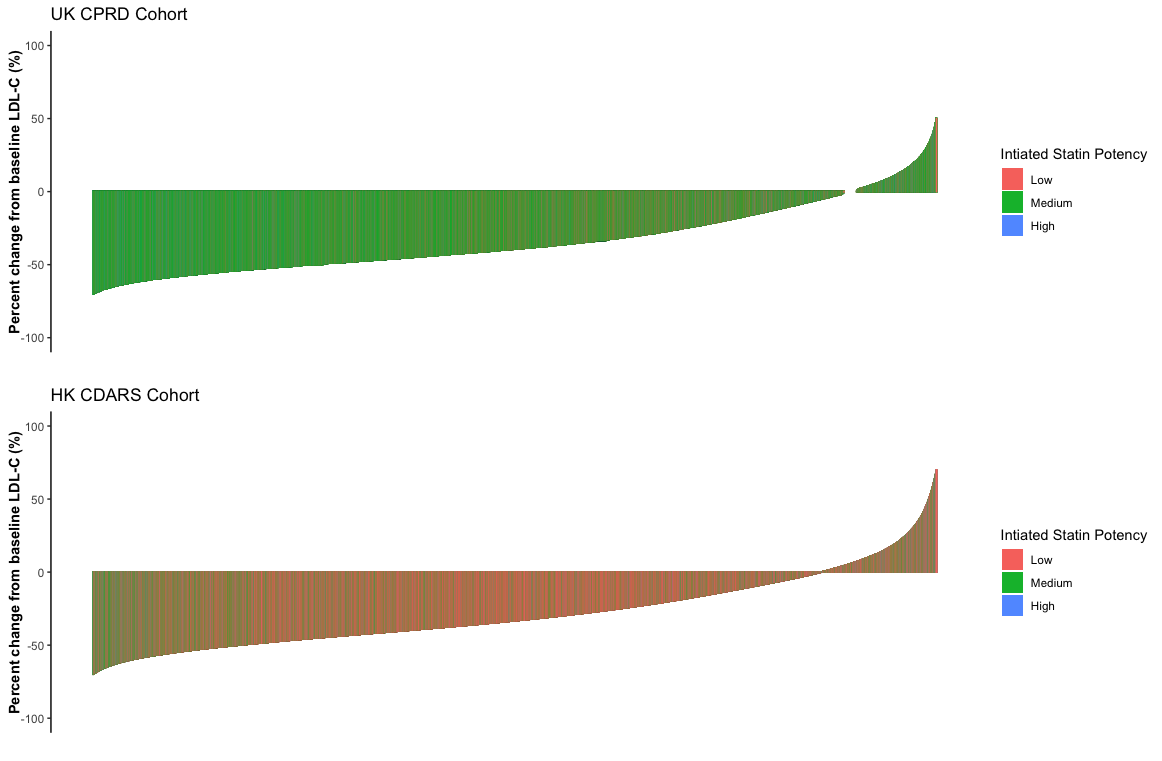
**

Potency classification by expected LDL-C reduction (see **S2 Table** for specific statin types)

20%–30%: low potency

31%–40%: medium potency

Above 40%: high potency
