# Supplementary material for: Determining propensity for sub-optimal low-density lipoprotein cholesterol response to statins and future risk of cardiovascular disease
Source: PLoS One. 2021 Dec 2;16(12):e0260839. doi: 10.1371/journal.pone.0260839 (PMC8638964; doi:10.1371/journal.pone.0260839)
Supplement: S1 Table — The choice of medications of medications is informed by review of clinical guidelines and previous literature on CVD risk (UK NICE Guidelines, QRISK3). (DOCX) [file pone.0260839.s006.docx]

**S1 Table. Potential predictors of sub-optimal statin response propensity model**

| - Age at study entry (baseline) - Gender (male or female) - LDL cholesterol (baseline) - HDL cholesterol (baseline) - Body mass index (BMI) - Systolic blood pressure (single most recent value at baseline) - Diastolic blood pressure (single most recent value at baseline) - Alcohol misuse - Smoking status (non-smoker; ex-smoker; smoker; unknown) - Atrial fibrillation (AF) - Chronic kidney disease - Diabetes - History of dyslipidaemia (hyperlipidaemia, hyperapobetalipoproteinemia, hypertriglyceridemia, hypercholesterolaemia – either familial or combined) - Family history of cardiovascular disease (CVD) - Family history of hyperlipidaemia - Treated hypertension (diagnosis of hypertension and a treatment with at least one antihypertensive medication within 12 months prior to entry date) - Hypothyroidism - Liver disease - Migraine - Nephrotic syndrome - Rheumatoid arthritis - Severe mental illness - Systemic lupus erythematosus - Medication count (at baseline – within 12 months prior to entry date) - Prescription of other lipid lowering medications (including fibrates, bile sequestrants, nicotinic acid, ezetimibe, or PCSK9 inhibitors at baseline – within 12 months prior to entry date) - Prescription of corticosteroids (at baseline – within 12 months prior to entry date) - Prescription of antipsychotics (within 12 months prior to entry date) - Potency of initial prescribed statin (low, medium, high) |
| --- |

The choice of medications of medications is informed by review of clinical guidelines and previous literature on CVD risk (UK NICE Guidelines, QRISK3).
