# Supplementary material for: Determining propensity for sub-optimal low-density lipoprotein cholesterol response to statins and future risk of cardiovascular disease
Source: PLoS One. 2021 Dec 2;16(12):e0260839. doi: 10.1371/journal.pone.0260839 (PMC8638964; doi:10.1371/journal.pone.0260839)
Supplement: S2 Table — 1 20%–30%: low intensity; 2 31%–40%: medium intensity; 3 Above 40%: high intensity. Note: combination therapies with a statin and ezetimibe 10 mg would push potency up one group (i.e., low to medium, medium to high). (DOCX) [file pone.0260839.s007.docx]

**S2 Table. Grouping of statins by potency**

Statins were grouped into three different intensity categories according to the percentage reduction in low‑density lipoprotein cholesterol from information provided UK National Institute for Health and Care Excellence (NICE) Clinical Guideline based on Law et al. 2003^2^.

|  | **Reduction in low‑density lipoprotein cholesterol** | | | | |
| --- | --- | --- | --- | --- | --- |
| **Dose (mg/day)** | 5 | 10 | 20 | 40 | 80 |
| Fluvastatin | – | – | 21%^1^ | 27%^1^ | 33%^2^ |
| Pravastatin | – | 20%^1^ | 24%^1^ | 29%^1^ | – |
| Simvastatin | – | 27%^1^ | 32%^2^ | 37%^2^ | 42%^3,4^ |
| Atorvastatin | – | 37%^2^ | 43%^3^ | 49%^3^ | 55%^3^ |
| Rosuvastatin | 38%^2^ | 43%^3^ | 48%^3^ | 53%^3^ | – |
| ^1^ 20%–30%: low intensity  ^2^ 31%–40%: medium intensity  ^3^ Above 40%: high intensity  Note: combination therapies with a statin and ezetimibe 10 mg would push potency up one group (i.e. low to medium, medium to high) | | | | | |
