# Supplementary material for: Determining propensity for sub-optimal low-density lipoprotein cholesterol response to statins and future risk of cardiovascular disease
Source: PLoS One. 2021 Dec 2;16(12):e0260839. doi: 10.1371/journal.pone.0260839 (PMC8638964; doi:10.1371/journal.pone.0260839)
Supplement: S3 Table — p < 0.05; No–reference category for dichotomous variables. (DOCX) [file pone.0260839.s008.docx]

# S3 Table. Multivariable logistic regression for mutually adjusted diagnostic variables of sub-optimal LDL-C response to statins using UK CPRD derivation cohort (n = 128,248)

| **Variables** | **Adjusted odds ratios (95% confidence interval)** | |
| --- | --- | --- |
|  | **Men** | **Women** |
| Age at time of statin prescription | 1.01 (0.99 – 1.04) | 1.00 (1.00 – 1.03) |
| Atrial fibrillation (AF) |  |  |
| Yes | 0.90 (0.83 – 0.99)* | 0.93 (0.84 – 1.03) |
| Diabetes |  |  |
| Yes | 0.66 (0.63 – 0.69)* | 0.69 (0.66 – 0.73)* |
| Diagnosed with lipid disorder (dyslipidaemias) |  |  |
| Yes | 3.24 (2.26 – 4.64)* | 2.69 (1.84 - 3.95)* |
| Potency of initial statin |  |  |
| Low | Reference | Reference |
| Medium | 0.65 (0.51 – 0.81)* | 0.85 (0.67 – 1.08) |
| High | 1.65 (1.4 – 2.39)* | 1.78 (1.16 – 2.73)* |
| Currently being treated for hypertension (Treated hypertension) |  |  |
| Yes | 0.52 (0.42 – 0.65)* | 0.41 (0.32 – 0.52)* |
| Prescription of corticosteroids (within the last 12 months) |  |  |
| Yes | 1.20 (1.10 – 1.32)* | 1.15 (1.07 – 1.25)* |
| Prescription of other lipid lowering medication, within last 12 months |  |  |
| Yes | 0.30 (0.12 – 0.79)* | 0.27 (0.07 – 1.03) |
| LDL-C level at time of statin prescription | 0.11 (0.09 – 0.12)* | 0.09 (0.08 – 0.11)* |
| Number of medications currently prescribed, including statin being considered (within last 12 months) | 0.78 (0.69 – 0.89)* | 0.91 (0.79 – 1.05) |
| **Interactions** | | |
| Age and medication count | 1.00 (1.00 – 1.00) | 1.00 (1.00 – 1.00) |
| Baseline LDL cholesterol level and dyslipidaemias | 0.51 (0.40 – 0.65)* | 0.61 (0.47 – 0.78)* |
| Potency of initial statin and baseline LDL cholesterol level |  |  |
| Low | Reference | Reference |
| Medium | 0.82 (0.69 – 0.96)* | 0.66 (0.56 – 0.77)* |
| High | 0.34 (0.26 – 0.44)* | 0.36 (0.27 – 0.49)* |
| Potency of initial statin and treated hypertension |  |  |
| Low | Reference | Reference |
| Medium | 0.89 (0.81 – 0.97)* | 0.94 (0.86 – 1.02) |
| High | 0.63 (0.52 – 0.77)* | 0.79 (0.65 – 0.96)* |
| Treated hypertension and baseline LDL cholesterol level | 1.51 (1.30 – 1.76)* | 1.78 (1.53 – 2.08)* |
| Prescription of other lipid lowering medication and baseline LDL cholesterol | 2.86 (1.38 – 5.95)* | 3.51 (1.34 – 9.25)* |
| * p < 0.05  No – reference category for dichotomous variables | | |
