# Supplementary material for: Determining propensity for sub-optimal low-density lipoprotein cholesterol response to statins and future risk of cardiovascular disease
Source: PLoS One. 2021 Dec 2;16(12):e0260839. doi: 10.1371/journal.pone.0260839 (PMC8638964; doi:10.1371/journal.pone.0260839)
Supplement: S4 Table — Unit: per mmol/L. (DOCX) [file pone.0260839.s009.docx]

**S4 Table. Logistic regression coefficients derived from the UK Clinical Practice Research Datalink (CPRD) for determining sub-optimal LDL-C response to statins**

| **Variables** | **Coefficients** | |
| --- | --- | --- |
|  | **Men** | **Women** |
| Atrial fibrillation (AF) |  |  |
| No | 0.0000000 | Reference |
| Yes | -0.1007224 | -0.0706856 |
| Diabetes |  |  |
| No | 0.0000000 | 0.0000000 |
| Yes | -0.4164963 | -0.3655665 |
| Dyslipidaemias |  |  |
| No | 0.000000 | 0.000000 |
| Yes | 1.175327 | 0.9901382 |
| Potency of initial statin |  |  |
| Low | 0.0000000 | 0.0000000 |
| Medium | -0.4348702 | -0.1633835 |
| High | 0.5023867 | 0.5785714 |
| Treated hypertension |  |  |
| No | 0.0000000 | Reference |
| Yes | -0.6556797 | -0.8930401 |
| Prescription of corticosteroids |  |  |
| No | 0.0000000 | 0.0000000 |
| Yes | 0.1856038 | 0.1424586 |
| Prescription of other lipid lowering medication |  |  |
| No | 0.000000 | 0.000000 |
| Yes | -1.188618 | -1.281212 |
| Baseline LDL cholesterol level* | -2.237247 | -2.402912 |
| Medication count | -0.2462398 | -0.0976184 |
| **Fractional polynomial transformed** | | |
| Age (3 3) |  |  |
| Age - 1 | -0.0000473 | -0.0000389 |
| Age -2 | 0.00000966 | 0.00000814 |
| **Interactions** | | |
| Age | 0.0140144 | 0.0002873 |
| Age and medication count | 0.0025746 | 0.0011338 |
| Baseline LDL cholesterol level* and dyslipidaemias | -0.6787666 | -0.4933142 |
| Potency of initial statin and baseline LDL cholesterol level* |  |  |
| Low | 0.0000000 | 0.0000000 |
| Medium | -0.2045539 | -0.4201863 |
| High | -1.087112 | -1.011652 |
| Potency of initial statin and treated hypertension |  |  |
| Low | 0.0000000 | 0.0000000 |
| Medium | -0.118732 | -0.0655084 |
| High | -0.4576589 | -0.2315173 |
| Treated hypertension and baseline LDL cholesterol level* | 0.4147712 | 0.5791071 |
| Prescription of other lipid lowering medication and baseline LDL cholesterol* | 1.050945 | 1.256923 |
| Constant term | 4.771014 | 5.286414 |

**unit: per mmol/L**
