# Supplementary material for: Determining propensity for sub-optimal low-density lipoprotein cholesterol response to statins and future risk of cardiovascular disease
Source: PLoS One. 2021 Dec 2;16(12):e0260839. doi: 10.1371/journal.pone.0260839 (PMC8638964; doi:10.1371/journal.pone.0260839)
Supplement: S5 Table — + Significance determined by Kruskal-Wallis non-parametric H test between two or more groups; ESC: European Society of Cardiology; CPRD: Clinical Practice Research Datalink; HDL: high-density lipoprotein; LDL: low-density lipoprotein; CVD: cardiovascular disease; SR1 –Patients with predicted optimal statin response and low CVD risk; SR2 –Patients with predicted sub-optimal statin response and low CVD risk; SR3 –Patients with predicted optimal statin response and high CVD risk; SR4 –Patients with predicted sub-optimal statin response and high CVD risk. (DOCX) [file pone.0260839.s010.docx]

**S5 Table. Characteristics of 183,213 patients from the UK CPRD dataset by statin response group based on ESC Score**

|  | Units | SR1  (n = 45,684; 24.9%) | SR2  (n = 64,997; 35.5%) | SR3  (n = 48,460; 26.5%) | SR4  (n = 24,072; 13.1%) | P-Value^†^ |
| --- | --- | --- | --- | --- | --- | --- |
| Age (Years) | Mean (SD)  Median (IQR) | 57.8 (8.1)  59.0 (53.0 -63.0) | 54.9 (9.7)  56.0 (49.0-62.0) | 73.2 (7.2)  73.0 (68.0-78.0) | 73.3 (7.2)  73.0 (68.0-78.0) | 0.0001 |
| Sex | No. (%) |  |  |  |  | < 0.001 |
| Male |  | 18,942 (41.5) | 36,167 (55.6) | 25,492 (52.6) | 15,560 (64.6) |  |
| Female |  | 26,742 (58.5) | 28,830 (44.4) | 22,968 (47.4) | 8,512 (35.4) |  |
| Ethnicity | No. (%) |  |  |  |  | < 0.001 |
| White |  | 20,460 (44.8) | 29,046 (44.7) | 25,804 (53.3) | 17,795 (53.2) |  |
| Non-white |  | 2,567 (5.6) | 5,165 (8.0) | 2,005 (4.1) | 1,092 (4.5) |  |
| Unknown |  | 22,657 (49.6) | 30,786 (47.4) | 20,651 (42.6) | 10,185 (42.3) |  |
| Systolic blood pressure (mmHg) | Mean (SD)  Median (IQR) | 138 (16)  138 (129-146) | 136 (16)  135 (126-144) | 151 (19)  149 (140-161) | 149 (19)  146 (138-160) | 0.0001 |
| Total cholesterol (mg/dL) | Mean (SD)  Median (IQR) | 267.6 (42.2)  266.8 (239.8-293.9) | 216.1 (42.1)  216.6 (1.89.5-243.6) | 258.6 (42.2)  255.2 (228.2-282.3) | 207.5 (39.3)  208.8 (181.7-234.0) | 0.0001 |
| HDL cholesterol (mg/dL) | Mean (SD)  Median (IQR) | 53.2 (15.5)  50.3 (42.5-61.1) | 51.9 (17.1)  49.1 (40.6-59.6) | 56.4 (16.2)  54.1 (46.4-65.0) | 56.2 (18.1)  53.4 (42.9-65.7) | 0.0001 |
| LDL cholesterol (mg/dL) | Mean (SD)  Median (IQR) | 187.6 (35.9)  182.5 (162.4-208.8) | 133.4 (36.8)  135.3 (111.0-158.5) | 174.9 (35.9)  170.1 (150.0-195.7) | 123.2 (33.1)  123.7 (100.5-146.9) | 0.0001 |
| Medication count | Median (IQR) | 5 (3-8) | 5 (3-8) | 6 (4-10) | 7 (4-10) | 0.0001 |
| History of diabetes | No. (%) | 8,563 (18.7) | 12,269 (18.9) | 6,452 (13.3) | 2,487 (10.3) | < 0.001 |
| Treated hypertension | No. (%) | 12,190 (26.7) | 12,004 (17.5) | 17,151 (35.4) | 6,605 (27.4) | < 0.001 |
| Smokers | No. (%) | 402 (0.9) | 714 (1.1) | 622 (1.3) | 488 (2.0) | < 0.001 |
| Statin potency | No. (%) |  |  |  |  | <0.001 |
| Low |  | 2,449 (5.4) | 22,245 (34.2) | 5,908 (12.2) | 17,702 (52.8) |  |
| Medium |  | 39,386 (86.2) | 39,479 (60.7) | 39,910 (82.4) | 10,486 (43.6) |  |
| High |  | 3,849 (8.4) | 3,273 (5.0) | 2,642 (5.5) | 884 (3.7) |  |

^+^ Significance determined by Kruskal-Wallis non-parametric H test between two or more groups; *ESC*: European Society of Cardiology; *CPRD*: Clinical Practice Research Datalink; *HDL*: high-density lipoprotein; *LDL*: low-density lipoprotein; *CVD*: cardiovascular disease; *SR1* – Patients with predicted optimal statin response and low CVD risk; *SR2* – Patients with predicted sub-optimal statin response and low CVD risk; *SR3* – Patients with predicted optimal statin response and high CVD risk; *SR4* – Patients with predicted sub-optimal statin response and high CVD risk.
