# Supplementary material for: Determining propensity for sub-optimal low-density lipoprotein cholesterol response to statins and future risk of cardiovascular disease
Source: PLoS One. 2021 Dec 2;16(12):e0260839. doi: 10.1371/journal.pone.0260839 (PMC8638964; doi:10.1371/journal.pone.0260839)
Supplement: S6 Table — + Significance determined by Kruskal-Wallis non-parametric H test between two or more groups; ESC: European Society of Cardiology; CPRD: Clinical Practice Research Datalink; HDL: high-density lipoprotein; LDL: low-density lipoprotein; CVD: cardiovascular disease; SR1 –Patients with predicted optimal statin response and low CVD risk; SR2 –Patients with predicted sub-optimal statin response and low CVD risk; SR3 –Patients with predicted optimal statin response and high CVD risk; SR4 –Patients with predicted sub-optimal statin response and high CVD risk. (DOCX) [file pone.0260839.s011.docx]

**S6 Table. Characteristics of 170,904 patients from the HK CDARS dataset by statin response group based on ESC Score**

|  | Units | SR1  (n = 47,842; 27.9%) | SR2  (n = 61,725; 36.1%) | SR3  (n = 43,514; 25.5%) | SR4  (n = 17,823, 10.4%) | P-Value^†^ |
| --- | --- | --- | --- | --- | --- | --- |
| Age (Years) | Mean (SD)  Median (IQR) | 58.6 (8.4)  59.1 (53.8-64.2) | 55.0 (9.2)  55.5 (49.5-61.4) | 74.7 (6.9)  74.9 (70.3-79.3) | 73.6 (6.9)  73.6 (69.2-78.1) | 0.0001 |
| Sex | No. (%) |  |  |  |  | < 0.001 |
| Male |  | 15,357 (32.1) | 32,885 (53.3) | 20,715 (47.6) | 12,632 (70.9) |  |
| Female |  | 32,485 (67.9) | 28,840 (46.7) | 22,799 (52.4) | 5,191 (29.1) |  |
| Total cholesterol (mg/dL) | Mean (SD)  Median (IQR) | 259.8 (41.9)  254.8 (233.2-278.4) | 207.4 (35.5)  206.9 (185.6-228.2) | 246.7 (42.8)  239.8 (220.4-266.8) | 191.7 (32.2)  191.8 (174.0-208.8) | 0.0001 |
| HDL cholesterol (mg/dL) | Mean (SD)  Median (IQR) | 51.8 (13.7)  49.9 (42.5-58.8) | 49.5 (14.2)  47.2 (39.4-56.8) | 51.3 (14.1)  49.1 (41.8-58.4) | 49.8 (15.1)  47.2 (39.1-57.6) | 0.0001 |
| LDL cholesterol (mg/dL) | Mean (SD)  Median (IQR) | 178.1 (35.8)  172.4 (156.3-193.3) | 127.1 (29.4)  128.4 (111.5-144.8) | 166.7 (36.4)  160.7 (144.0-182.0) | 113.6 (24.7)  116.7 (100.4-128.3) | 0.0001 |
| Medication count | Median (IQR) | 7(5-12) | 7(4-10) | 9(6-13) | 9(5-13) | 0.0001 |
| History of diabetes | No. (%) | 11,190 (23.4) | 8,777 (14.2) | 10,526 (24.2) | 1,964 (11.0) | < 0.001 |
| Treated hypertension | No. (%) | 6,936 (14.5) | 4,370 (7.1) | 9,571 (22.0) | 1,793 (10.1) | < 0.001 |
| Smokers | No. (%) | 616 (1.3) | 701 (1.1) | 2,373 (5.5) | 924 (5.2) | < 0.001 |
| Statin potency | No. (%) |  |  |  |  | < 0.001 |
| Low |  | 30,629 (64.0) | 43,253 (70.1) | 30,807 (70.8) | 12,418 (69.7) |  |
| Medium |  | 16,759 (35.0) | 14,616 (23.7) | 12,136 (27.9) | 4,314 (24.2) |  |
| High |  | 454 (1.0) | 3,856 (6.3) | 571 (1.3) | 1,091 (6.1) |  |

^+^ Significance determined by Kruskal-Wallis non-parametric H test between two or more groups; *ESC*: European Society of Cardiology; *CDARS*: Clinical Data Analysis and Reporting System; *HDL*: high-density lipoprotein; *LDL*: low-density lipoprotein; *CVD*: cardiovascular disease; *HK*: Hong Kong; *SR1* – Patients with predicted optimal statin response and low CVD risk; *SR2* – Patients with predicted sub-optimal statin response and low CVD risk; *SR3* – Patients with predicted optimal statin response and high CVD risk; *SR4* – Patients with predicted sub-optimal statin response and high CVD risk.
