# Supplementary material for: Determining propensity for sub-optimal low-density lipoprotein cholesterol response to statins and future risk of cardiovascular disease
Source: PLoS One. 2021 Dec 2;16(12):e0260839. doi: 10.1371/journal.pone.0260839 (PMC8638964; doi:10.1371/journal.pone.0260839)
Supplement: S7 Table — 95% Confidence Intervals are provided in () for all incidence rates. SR1 –Patients with predicted optimal statin response and low CVD risk; SR2 –Patients with predicted sub-optimal statin response and low CVD risk; SR3 –Patients with predicted optimal statin response and high CVD risk; SR4 –Patients with predicted sub-optimal statin response and high CVD risk. ESC: European Society of Cardiology; CPRD: Clinical Practice Research Datalink; CDARS: Clinical Data Analysis and Reporting System; CVD: cardiovascular disease; TIA: Transient Ischemic Attack; CHD: Coronary Heart Disease; PVD: Peripheral Vascular Disease; UK: United Kingdom; HK: Hong Kong. (DOCX) [file pone.0260839.s012.docx]

**S7 Table. 10-year incidence rates (per 1000 person-years) for major adverse cardiovascular events (MACE) and all-cause mortality for predicted statin response and ESC cardiovascular risk groups.**

| **Phenotypic Groups** | **All Major Adverse CVD Events** | **All-Cause Mortality** | **Specific CVD Outcomes** | | | |
| --- | --- | --- | --- | --- | --- | --- |
|  |  |  | CHD | Stroke/TIA | PVD | CVD Death |
| **United Kingdom CPRD Cohort** | 41.6 (41.2 – 41.9) | 13.4 (13.2 – 13.6) | 27.7 (27.3 – 28.0) | 7.1 (6.9 – 7.3) | 4.9 (4.8 – 5.0) | 1.8 (1.8 – 1.9) |
| SR1 | 26.1 (25.5 – 26.7) | 6.8 (6.5 – 7.1) | 18.4 (17.9 – 18.9) | 3.8 (3.6 – 4.0) | 3.0 (2.8 – 3.2) | 0.8 (0.7 – 0.9) |
| SR2 | 36.5 (35.9 – 37.1) | 6.4 (6.1 – 6.6) | 26.9 (26.4 – 27.5) | 4.9 (4.7 – 5.2) | 3.8 (3.6 – 4.1) | 0.7 (0.6 – 0.8) |
| SR3 | 50.8 (49.9 – 51.7) | 23.9 (23.3 – 24.5) | 29.9 (29.3 – 30.6) | 10.6 (10.2 – 11.0) | 6.8 (6.5 – 7.1) | 3.5 (3.3 – 3.7) |
| SR4 | 70.3 (68.8 – 71.8) | 26.0 (25.1 – 26.9) | 45.1 (43.9 – 46.3) | 13.3 (12.6 – 13.9) | 8.1 (7.6 – 8.7) | 3.8 (3.5 – 4.2) |
| **Hong Kong CDARS Cohort** | 48.0 (48.6 – 49.5) | 27.2 (26.9 – 27.5) | 16.8 (16.4 – 16.9) | 13.7 (13.5 – 13.9) | 1.5 (1.4 – 1.6) | 4.3 (4.2 – 4.5) |
| SR1 | 32.4 (31.8 – 33.0) | 14.1 (13.7 – 14.6) | 12.4 (12.1 – 12.8) | 10.6 (10.2 – 10.9) | 1.2 (1.0 – 1.3) | 2.6 (2.4 – 2.8) |
| SR2 | 37.4 (36.8 – 38.0) | 11.2 (10.9 – 11.5) | 18.9 (18.5 – 19.4) | 10.8 (10.5 – 11.1) | 1.1 (1.0 – 1.2) | 1.8 (1.7 – 1.9) |
| SR3 | 75.9 (74.7 – 76.9) | 60.2 (59.2 – 61.2) | 15.2 (14.7 – 15.7) | 20.2 (19.6 – 20.8) | 2.2 (2.1 – 2.5) | 9.4 (9.0 – 9.8) |
| SR4 | 95.4 (93.4 – 97.5) | 64.0 (62.4 – 65.8) | 26.0 (24.9 – 27.1) | 22.4 (21.4 – 23.5) | 2.6 (2.2 – 2.9) | 9.5 (8.8 – 10.1) |
| 95% Confidence Intervals are provided in ( ) for all incidence rates  *SR1* – Patients with predicted optimal statin response and low CVD risk; *SR2* – Patients with predicted sub-optimal statin response and low CVD risk; *SR3* – Patients with predicted optimal statin response and high CVD risk; *SR4* – Patients with predicted sub-optimal statin response and high CVD risk  ESC: European Society of Cardiology; CPRD: Clinical Practice Research Datalink; CDARS: Clinical Data Analysis and Reporting System; CVD: cardiovascular disease; TIA: Transient Ischemic Attack; CHD: Coronary Heart Disease; PVD: Peripheral Vascular Disease; UK: United Kingdom; HK: Hong Kong. | | | | | | |
