# Supplementary material for: Determining propensity for sub-optimal low-density lipoprotein cholesterol response to statins and future risk of cardiovascular disease
Source: PLoS One. 2021 Dec 2;16(12):e0260839. doi: 10.1371/journal.pone.0260839 (PMC8638964; doi:10.1371/journal.pone.0260839)
Supplement: S1 Text — (DOCX) [file pone.0260839.s013.docx]

**ONLINE SUPPLEMENTAL METHODS**

### Development and validation of statin response model

To develop the predictive model, a randomly selected sample of 128,248 (70%) patients from the CPRD study population was used as the derivation cohort. Candidate predictors (documented in **S1 Table**) were assessed for inclusion by clinical review and prior evidence. Backwards elimination based on minimising the Akaike Information Criterion (AIC) was conducted to determine best-fitting model. Fractional polynomials were used to model non-linear risk relations with continuous variables and interactions between risk factors were tested^1^. Multiple imputation with chained equations was used to estimate missing values for blood pressure (systolic and diastolic) and body mass index^2^. All other patient variables were included in the imputation models to create ten imputed datasets. Using Rubin’s rules, we pooled regression equation to determine the propensity (predicted probability) of sub-optimal LDL-C response to initiated statin.

The regression equations from the derivation cohort was applied to two validation cohorts to calculate each patient’s propensity for sub-optimal cholesterol response to initiated statin: internal validation using the remaining 30% random sample from the UK CPRD cohort (n = 54,965) and a true external validation using an entirely separate cohort from HK CDARS (n = 170,904). Discrimination was assessed using the area under the receiver operating curve (AUROC) or Harrell’s *c*-statistics; with higher values representing better discrimination. To generate confidence intervals for the *c*-statistics, a bootstrapping was used to estimate standard errors^3^. The degree of similarity between observed and predicted propensity of sub-optimal response, was assessed by a calibration plot as recommended by TRIPOD guidelines^4^. We assessed the sensitivity and specificity for the model at various propensity thresholds. For binary classification of sub-optimal SR, the intersection between sensitivity and specificity was determined as the optimal threshold, corresponding to the maximum AUROC^5^.

A sub-group analysis was performed to evaluate the impact of adherence on model performance, by developing a proxy marker based on medication possession counts (MPC) over two-year period from baseline using prescribing records in the UK validation cohort (due to having access to full UK prescribing records). Discontinuation periods were calculated based on pack size, pack numbers and daily dosage. The total number of days covered by prescriptions was estimated, factoring in periods of discontinuation, divided by the total number of days in the period, creating the proxy for statin adherence rate. We evaluated model performance in each 5 pre-specified sub-groups for adherence: <20%, 20-<40%, 40-<60%, 60-<80%, ≥80%.

### Clinical validity of stratified groups and future MACE

Patients were stratified into four groups based on calculated SR propensity and 10-year CVD risk (using European Society of Cardiology SCORE equations): [SR1] predicted optimal SR & low CVD risk; [SR2] predicted sub-optimal SR & low CVD risk; [SR3] predicted optimal SR & high CVD risk; [SR4] predicted sub-optimal SR & high CVD risk. ESC defines high risk of CVD for individuals as baseline CVD risk above 5% for consideration for initiation of statin therapy. ESC SCORE equations were used due to its simplicity in calculation (limited number of risk factors) and being acceptable for use UK and HK populations^6,7^.

Groups in both UK and HK cohorts were characterised, and their differences compared. Incidence rates for 10-year MACE and all-cause mortality were provided with 95% confidence intervals (CI). To obtain estimates for the association between predicted sub-optimal cholesterol response to statin therapy and incident MACE, we used Cox proportional hazards regression analysis, with informative censoring of the survival time when patients were lost to follow-up or died due to other causes (apart from the all-cause mortality model which included all outcomes related to death). These hazard models were stratified by low (≤ 5% baseline CVD risk) and high baseline CVD risk (> 5% baseline CVD risk).

To obtain cumulative incidence graphs of MACE for each phenotypic group, we used competing-risk analysis^8^ to determine the cause-specific hazard ratio in the presence of non-CVD related death acting as the competing event. All models were assessed for proportional hazards using Schoenfeld residuals^9^. P-values less than 0.05 were considered to be statistically significant.

1. Royston P, Ambler G, Sauerbrei W. The use of fractional polynomials to model continuous risk variables in epidemiology. *Int J Epidemiol*. 1999;28:964–974.

2. Royston P. Multiple imputation of missing values: update. *Stata J*. 2005;5:188–201.

3. Newson R. Confidence intervals for rank statistics: Somers’ D and extensions. *Stata J* . 2006;6:309–34.

4. Collins GS, Reitsma JB, Altman DG, Moons KGM. Transparent Reporting of a Multivariable Prediction Model for Individual Prognosis or Diagnosis (TRIPOD). *Circulation*. 2015;131:211–219.

5. Liu X. Classification accuracy and cut point selection. *Stat Med*. 2012;31:2676–2686.

6. Piepoli MF, Hoes AW, Agewall S, Albus C, Brotons C, Catapano AL, Cooney M-T, Corrà U, Cosyns B, Deaton C, Graham I, Hall MS, Hobbs FDR, Løchen M-L, Löllgen H, Marques-Vidal P, Perk J, Prescott E, Redon J, Richter DJ, Sattar N, Smulders Y, Tiberi M, van der Worp HB, van Dis I, Verschuren WMM, Binno S, De Backer G, Roffi M, Aboyans V, Bachl N, Bueno H, Carerj S, Cho L, Cox J, De Sutter J, Egidi G, Fisher M, Fitzsimons D, Franco OH, Guenoun M, Jennings C, Jug B, Kirchhof P, Kotseva K, Lip GYH, Mach F, Mancia G, Bermudo FM, Mezzani A, Niessner A, Ponikowski P, Rauch B, Rydén L, Stauder A, Turc G, Wiklund O, Windecker S, Zamorano JL, Zamorano JL, Aboyans V, Achenbach S, Agewall S, Badimon L, Barón-Esquivias G, Baumgartner H, Bax JJ, Bueno H, Carerj S, Dean V, Erol Ç, Fitzsimons D, Gaemperli O, Kirchhof P, Kolh P, Lancellotti P, Lip GYH, Nihoyannopoulos P, Piepoli MF, Ponikowski P, Roffi M, Torbicki A, Vaz Carneiro A, Windecker S, Metzler B, Najafov R, Stelmashok V, De Maeyer C, Dilic M, Gruev I, Milicic D, Vaverkova H, Gustafsson I, Attia I, Duishvili D, Kostova N, Ferrières J, Klimiashvili Z, Hambrecht R, Tsioufis K, Szabados E, Andersen K, Vaughan C, Zafrir B, Novo S, Davletov K, Jashari F, Kerimkulova A, Mintale I, Saade G, Petrulioniene Z, Delagardelle C, Magri CJ, Rudi V, Oukerraj L, Çölkesen BE, Schirmer H, Jankowski P, dos Reis RP, Gherasim D, Nedogoda S, Zavatta M, Giga V, Filipova S, Padial LR, Kiessling A, Mach F, Mahdhaoui A, Ural D, Nesukay E, Gale C. 2016 European Guidelines on cardiovascular disease prevention in clinical practice. *Eur Heart J*. 2016;37:2315–2381.

7. Cheung BM, Cheng CH, Lau CP, Wong CK, Ma RC, Chu DW, Ho DH, Lee KL, Tse HF, Wong AS, Yan BP, Yan VW. 2016 Consensus statement on prevention of atherosclerotic cardiovascular disease in the Hong Kong population. *Hong Kong Med J*. 2017;23:191–201.

8. Austin PC, Lee DS, Fine JP. Introduction to the Analysis of Survival Data in the Presence of Competing Risks. *Circulation*. 2016;133:601–609.

9. Hess KR. Graphical methods for assessing violations of the proportional hazards assumption in Cox regression. *Stat Med*. 1995;14:1707–1723.
