# Supplementary material for: Determining propensity for sub-optimal low-density lipoprotein cholesterol response to statins and future risk of cardiovascular disease
Source: PLoS One. 2021 Dec 2;16(12):e0260839. doi: 10.1371/journal.pone.0260839 (PMC8638964; doi:10.1371/journal.pone.0260839)
Supplement: Attachment — Submitted filename: Response to Reviewers.docx [file pone.0260839.s014.docx]

***Journal:***	*PLOS ONE*

***Manuscript #:***	*PONE-D-21-11502R1*

***Title of Paper:***	*Determining propensity for sub-optimal low-density lipoprotein cholesterol response to statins and future risk of cardiovascular disease*

***Authors:***	*Stephen F Weng, Ralph K Akyea, Kenneth KC Man, Wallis CY Lau, Barbara Iyen, Joseph Edgar Blais, Esther W Chan, Chung Wah Siu, Nadeem Qureshi, Ian CK Wong, Joe Kai*

We greatly appreciate the overall assessment and feedback by the Editorial Committee and Reviewers. We have revised our manuscript in the light of your helpful comments. Our response to each of the comments and recommendations are detailed below.

**Response to Comments from Editorial Committee**

Comment:

*1. Please ensure that your manuscript meets PLOS ONE's style requirements, including those for file naming. The PLOS ONE style templates can be found at*

[*https://journals.plos.org/plosone/s/file?id=wjVg/PLOSOne_formatting_sample_main_body.pdf*](https://journals.plos.org/plosone/s/file?id=wjVg/PLOSOne_formatting_sample_main_body.pdf) *and*

[*https://journals.plos.org/plosone/s/file?id=ba62/PLOSOne_formatting_sample_title_authors_affiliations.pdf*](https://journals.plos.org/plosone/s/file?id=ba62/PLOSOne_formatting_sample_title_authors_affiliations.pdf)

Response:

The manuscript has been formatted/revised to meet PLOS ONE’s style requirements, including those for file naming.

Comment:

*2. Thank you for stating the following in the Acknowledgments Section of your manuscript:*

*"JB is supported by the Hong Kong Research Grants Council as a recipient of the Hong Kong*

*PhD Fellowship Scheme."*

*We note that you have provided funding information that is not currently declared in your Funding Statement. However, funding information should not appear in the Acknowledgments section or other areas of your manuscript. We will only publish funding information present in the Funding Statement section of the online submission form.*

*Please remove any funding-related text from the manuscript and let us know how you would like to update your Funding Statement. Currently, your Funding Statement reads as follows:*

*Please include your amended statements within your cover letter; we will change the online submission form on your behalf.*

Response:

The funding statement "JB is supported by the Hong Kong Research Grants Council as a recipient of the Hong Kong PhD Fellowship Scheme" has been removed from the acknowledgement section to the competing interests/financial disclosure section.

The funding statement remains the same: “Funded by scientific donation by AMGEN Ltd. The funders of the study had no role in study design, data collection, data analysis, data interpretation, or writing of the report”.

Comment:

*3. Thank you for stating the following in the Competing Interests/Financial Disclosure * (delete as necessary) section:*

*"Funded by scientific donation by AMGEN Ltd. The funders of the study had no role in study design, data collection, data analysis, data interpretation, or writing of the report."*

*We note that you received funding from a commercial source: AMGEN Ltd.*

*Please provide an amended Competing Interests Statement that explicitly states this commercial funder, along with any other relevant declarations relating to employment, consultancy, patents, products in development, marketed products, etc.*

*Within this Competing Interests Statement, please confirm that this does not alter your adherence to all PLOS ONE policies on sharing data and materials by including the following statement: "This does not alter our adherence to PLOS ONE policies on sharing data and materials.” (as detailed online in our guide for authors* [*http://journals.plos.org/plosone/s/competing-interests*](http://journals.plos.org/plosone/s/competing-interests)*). If there are restrictions on sharing of data and/or materials, please state these. Please note that we cannot proceed with consideration of your article until this information has been declared.*

*Please include your amended Competing Interests Statement within your cover letter. We will change the online submission form on your behalf.*

*Please know it is PLOS ONE policy for corresponding authors to declare, on behalf of all authors, all potential competing interests for the purposes of transparency. PLOS defines a competing interest as anything that interferes with, or could reasonably be perceived as interfering with, the full and objective presentation, peer review, editorial decision-making, or publication of research or non-research articles submitted to one of the journals. Competing interests can be financial or non-financial, professional, or personal. Competing interests can arise in relationship to an organization or another person. Please follow this link to our website for more details on competing interests:* [*http://journals.plos.org/plosone/s/competing-interests*](http://journals.plos.org/plosone/s/competing-interests)

Response:

The competing interests/financial disclosure section has been revised accordingly and now includes the statement "This does not alter our adherence to PLOS ONE policies on sharing data and materials.”, as required. The competing interest statement now reads (page 22):

*“NQ is a member of the most recent NICE Familial Hypercholesterolaemia & Lipid Modification Guideline Development Groups (CG71 & CG181). SW, IW are members of the Clinical Practice Research Datalink (CPRD) Independent Scientific Advisory Committee (ISAC). RKA currently holds an NIHR-SPCR funded studentship (2018-2021). SW reports honorarium from AMGEN and is also an employee of Janssen. KM holds the CW Malpethorpe Fellowship Award and reports personal fees from IQVIA Ltd have been received outside the submitted work. JB is supported by the Hong Kong Research Grants Council as a recipient of the Hong Kong PhD Fellowship Scheme. EC and IW report grants from Amgen. The remaining authors have no competing interests. This does not alter our adherence to PLOS ONE policies on sharing data and materials.”*

Comment:

*4. Thank you for stating the following in the Competing Interests section:*

*"NQ is a member of the most recent NICE Familial Hypercholesterolaemia & Lipid Modification Guideline Development Groups (CG71 & CG181). SW, IW are members of the Clinical Practice Research Datalink (CPRD) Independent Scientific Advisory Committee (ISAC). RKA currently holds an NIHR-SPCR funded studentship (2018-2021). SW reports honorarium from AMGEN and is also an employee of Janssen. KM holds the CW Malpethorpe Fellowship Award and reports personal fees from IQVIA Ltd have been received outside the submitted work. EC and IW report grants from Amgen. The remaining authors have no competing interests."*

*Please confirm that this does not alter your adherence to all PLOS ONE policies on sharing data and materials, by including the following statement: "This does not alter our adherence to PLOS ONE policies on sharing data and materials.” (as detailed online in our guide for authors* [*http://journals.plos.org/plosone/s/competing-interests*](http://journals.plos.org/plosone/s/competing-interests)*). If there are restrictions on sharing of data and/or materials, please state these. Please note that we cannot proceed with consideration of your article until this information has been declared.*

*Please include your updated Competing Interests statement in your cover letter; we will change the online submission form on your behalf.*

*Please know it is PLOS ONE policy for corresponding authors to declare, on behalf of all authors, all potential competing interests for the purposes of transparency. PLOS defines a competing interest as anything that interferes with, or could reasonably be perceived as interfering with, the full and objective presentation, peer review, editorial decision-making, or publication of research or non-research articles submitted to one of the journals. Competing interests can be financial or non-financial, professional, or personal. Competing interests can arise in relationship to an organization or another person. Please follow this link to our website for more details on competing interests:* [*http://journals.plos.org/plosone/s/competing-interests*](http://journals.plos.org/plosone/s/competing-interests)

Response:

As indicated in the response to the earlier comment, the competing interest section has been revised as required and the sentence “The remaining authors have no competing interests. This does not alter our adherence to PLOS ONE policies on sharing data and materials.” has been added (page 20).

Comment:

*5. We note that you have indicated that data from this study are available upon request. PLOS only allows data to be available upon request if there are legal or ethical restrictions on sharing data publicly. For information on unacceptable data access restrictions, please see* [*http://journals.plos.org/plosone/s/data-availability#loc-unacceptable-data-access-restrictions*](http://journals.plos.org/plosone/s/data-availability#loc-unacceptable-data-access-restrictions)*.*

*In your revised cover letter, please address the following prompts:*

*a) If there are ethical or legal restrictions on sharing a de-identified data set, please explain them in detail (e.g., data contain potentially identifying or sensitive patient information) and who has imposed them (e.g., an ethics committee). Please also provide contact information for a data access committee, ethics committee, or other institutional body to which data requests may be sent.*

*b) If there are no restrictions, please upload the minimal anonymized data set necessary to replicate your study findings as either Supporting Information files or to a stable, public repository and provide us with the relevant URLs, DOIs, or accession numbers. Please see http://www.bmj.com/content/340/bmj.c181.long for guidelines on how to de-identify and prepare clinical data for publication. For a list of acceptable repositories, please see* [*http://journals.plos.org/plosone/s/data-availability#loc-recommended-repositories*](http://journals.plos.org/plosone/s/data-availability#loc-recommended-repositories)*.*

*We will update your Data Availability statement on your behalf to reflect the information you provide.*

Response:

There are ethical and legal restrictions on sharing de-identified data set and this has been explained in the revised data availability section (page 22):

*“The third-party data for this study were obtained from the Clinical Practice Research Datalink (CPRD) and the University of Hong Kong/Hospital Authority Hong Kong West Cluster. CPRD is a research service that provides primary care and linked data for public health research. CPRD and University of Hong Kong/Hospital Authority data governance and our own license to use data do not allow us to distribute or make available patient data directly to other parties. However, data is available upon application to CPRD (*[*www.cprd.com*](http://www.cprd.com)*) and the University of Hong Kong/Hospital Authority (*[*www.ha.org.hk*](http://www.ha.org.hk)*).”*

**Response to Comments from Reviewer 1**

**Specific comments**

Comment:

*“I’ve read with attention the paper of Ralph Kwame Akyea et al. that is potentially of interest. The background and aim of the study have been clearly defined. The methodology applied is overall correct and the results are reliable. I’ve only some minor comments: - The difference in use of low intensity statin in the two cohort could be influenced by the fact that Asian are more sensible to statin-side effects and some drugs are to be used at lower dosages in Asians”*

Response:

We thank the reviewer for the positive assessment.

Comment:

*“The authors stress that ezetimibe should be used in secondary prevention patients only... why? The guidelines suggest adding ezetimibe where LDL-C target is not achieved, in high-risk patients in primary prevention as well”*

Response:

Thank you for highlighting this. We agree with the reviewer that the guidelines recommend Ezetimibe for primary prevention as well. The sentence has, therefore, been revised to reflect this. The sentence now reads (page 14): “Medications that aim to reduce LDL-C such as PCSK9 inhibitors and Ezetimibe, [26,27] are presently indicated for both primary and secondary prevention of CVD.”

Comment:

*“The authors suggest the use of icosapent ethyl as an adjunct to diet in adults with triglycerides of at least 500 mg/dL. This is not supported by the reference chosen by the authors, nor it is supported by any guideline. Probably fenofibrate should be considered as well.”*

Response:

The paragraph have been revised and the bit about the use of icosapent ethyl as an adjunct to diet in adults with triglycerides of at least 500mg/dL, has been removed. The paragraph now reads (page 14): “Medications that aim to reduce LDL-C such as PCSK9 inhibitors and Ezetimibe, [26,27] are presently indicated for both primary and secondary prevention of CVD. When taken in combination with statin and diet therapies, additional reduction in LDL-C levels have been shown in trials.”
